# The association between adverse pregnancy outcomes and non-viral genital pathogens among women living in sub-Saharan Africa: a systematic review

**DOI:** 10.3389/frph.2023.1107931

**Published:** 2023-06-07

**Authors:** Carlotta Gamberini, Naomi C. A. Juliana, Lenya de Brouwer, Dorothea Vogelsang, Salwan Al-Nasiry, Servaas A. Morré, Elena Ambrosino

**Affiliations:** ^1^ Institute for Public Health Genomics (IPHG), Department of Genetics and Cell Biology, Research School GROW for Oncology and Reproduction, Faculty of Health, Medicine & Life Sciences, University of Maastricht, Maastricht, Netherlands; ^2^ Research School GROW for Oncology and Reproduction, Maastricht University, Maastricht, Netherlands; ^3^ Department of Obstetrics and Gynecology, Research School GROW for Oncology and Reproduction, Maastricht University Medical Centre+, Maastricht, Netherlands; ^4^ Department of Molecular and Cellular Engineering, Jacob Institute of Biotechnology and Bioengineering, Sam Higginbottom University of Agriculture, Technology and Sciences, Allahabad, UP, India; ^5^ Dutch Chlamydia trachomatis Reference Laboratory on Behalf of the Epidemiology and Surveillance Unit, Centre for Infectious Disease Control, National Institute for Public Health and the Environment, Bilthoven, Netherlands

**Keywords:** non-viral genital pathogens, chlamydia trachomatis (CT), treponema pallidum (TP), candida albicans, antenatal care (ANC), pregnancy outcomes

## Abstract

Adverse pregnancy outcomes are the main causes of maternal and neonatal morbidity and mortality, including long-term physical and psychological sequelae. These events are common in low- and middle-income countries, particularly in Sub Saharan Africa, despite national efforts. Maternal infections can cause complications at any stage of pregnancy and contribute to adverse outcomes. Among infections, those of the genital tract are a major public health concern worldwide, due to limited availability of prevention, diagnosis and treatment approaches. This applies even to treatable infections and holds true especially in Sub-Saharan Africa. As late as 2017, the region accounted for 40% of all reported treatable non-viral genital pathogens worldwide, many of which have been independently associated with various adverse pregnancy outcomes, and that include *Chlamydia trachomatis*, *Neisseria gonorrhoeae*, *Trichomonas vaginalis*, *Treponema pallidum*. Two databases (PubMed and Embase) were examined to identify eligible studies published up to October 2022. This study reviewed findings on the association between infections by treatable non-viral genital pathogens during pregnancy and adverse pregnancy outcomes among women living in Sub-Saharan Africa. Articles' title and abstract were screened at first using keywords as “sexually transmitted infections”, “non-viral”, “adverse pregnancy outcome”, “Africa”, “sub-Saharan Africa”, “pregnant women”, “pregnancy”, and “pregnancy outcome”. Subsequently, according to the eligibility criteria, potential articles were read in full. Results showed that higher risk of preterm birth is associated with *Treponema pallidum, Chlamydia trachomatis* and *Candida albicans* infections*.* Additionally, rates of stillbirth, neonatal death, low birth weight and intrauterine growth restriction are also associated with *Treponema pallidum* infection*.* A better insight on the burden of non-viral genital pathogens and their effect on pregnancy is needed to inform antenatal care guidelines and screening programs, to guide the development of innovative diagnostic tools and other strategies to minimize transmission, and to prevent short- and long-term complications for mothers and children.

## Introduction

Every day, approximately 810 women die from preventable complications during pregnancy, childbirth, or the postpartum period, cording to the 2019 Lancet global report, approximately 2.9 million newborns die in their first month of life, with caccording to the World Health Organization (1). Acomplications in pregnancy being the leading causes of death and other adverse pregnancy outcomes (2, 3). Adverse pregnancy outcomes are the main cause of maternal and neonatal morbidity and mortality and long-term physical and psychological sequelae in low- and middle-income countries (LMICs), with the most common being low birth weight (LBW), preterm birth (PTB), stillbirth, or perinatal death (4, 5). Factors associated with adverse pregnancy outcomes include intra- or extra-uterine infections, microbial dysbiosis, and an abnormal immune system, among others. Maternal infections caused by bacteria, viruses, parasites, or fungi can cause complications at any stage of pregnancy. Several studies have found that pregnant women are more susceptible to various infections due to compensatory physiological and immunologic responses (6, 7).

Non-viral genital pathogens are often easily treatable, but if not managed during pregnancy, they have been associated with poor pregnancy outcomes. Caused by more than 30 different microorganisms, non-viral genital infections spread through unprotected sexual contact or, in the case of pregnant women, also vertically from an infected mother to the child, and are a growing public health concern worldwide (8). The WHO estimates that around 500 million people around the globe become infected with one of curable, non-viral pathogens (*Chlamydia trachomatis*, *Neisseria gonorrhoeae, Trichomonas vaginalis, Treponema pallidum,* and *Mycoplasma genitalium*) yearly. If these infections are left untreated, they would cause serious pregnancy complications which could impact the female reproductive system, such as pelvic inflammatory disease, cervicitis, neurological problems, cardiovascular problems, infertility and/or ectopic pregnancy (9–12). Sub-Saharan Africa bears 40% of this global burden, with women frequently being infected more often than men (12). The burden of common non-viral genital pathogens remains high in sub-Saharan Africa among expectant mothers. Between 2000 and 2011, the prevalence during pregnancy of *Treponema pallidum* infection in sub-Saharan Africa was 2.9% (2.1%–3.6%), *Neisseria gonorrhoeae* was 4.9% (1.8%–7.9%), *Chlamydia trachomatis* was 5.2% (3.4%–7.1%) and *Trichomonas vaginalis* was 24.9% (18.3%–31.5%) in East and Southern Africa (13). In West and Central Africa, the prevalence was lower; the incidence of *T. pallidum* infection was 2.5% (0.4%–4.6%), of *N. gonorrhoeae* infection was 1.6% (0.0%–3.3%), of *C. trachomatis* infection was 1.9% (0.2%–3.5%) and that of *T. vaginalis* infection was 4.5% (2.5%–6.6%) among pregnant women (13).

Because of the high burden of viral sexually transmitted infections (STIs) in sub-Saharan Africa and the known consequences that these viral pathogens have on maternal and child's health, a large number of resources has been dedicated to address viral sexually transmitted infections during pregnancy. Sexual and nonsexual transmission of these viral pathogens, such as Human Immunodeficiency Virus and Human Papilloma Virus, are known to cause infections, diseases and long-term health consequences (14–17). However, non-viral genital pathogens remain a relatively neglected area of research, despite their high global burden, the advantage of being curable and the serious consequences that untreated infection can have.

Genital infections and pregnancy-related complications are examples of traditionally overlooked health challenges that continue to carry a significant burden on women's health (18). Because of the frequent absence of laboratory tests in rural areas, where most of the population resides in the sub-Saharan African region, diagnosis and treatment of infections is frequently based on signs and symptoms (syndromic management). However, non-viral genital pathogens are often asymptomatic and remain undiagnosed, untreated or treated with a significant delay (19, 20). Most non-viral genital pathogens are curable and do not influence pregnancy outcomes if treated in time (21); conversely, if left untreated, serious late adverse pregnancy outcomes can occur, including LBW, PTB, stillbirth, neonatal death or maternal mortality and morbidity (21–25). It has been estimated that almost 98% of 2.6 million stillbirths that occur annually in LMICs, most of which in sub-Saharan Africa, are associated with genital infections, and could be prevented (1, 12, 26, 27). Earlier studies have provided prevalence of several non-viral genital pathogens in sub-Saharan Africa (9, 13, 28, 29), including that by Mullick et al., which broadly reviewed in 2005 the prevalence of sexually transmitted infections, their impact on pregnancy outcomes, and the approach to treat them in pregnancy in both LMICs and high-income countries (HICs) (30). However, evidence remains scant and to our knowledge, no up to date comprehensive systematic review has focused on the association between non-viral genital pathogens and adverse pregnancy outcomes in sub-Saharan Africa in over a decade. This study comprehensively and systematically reviewed published data on the association between non-viral genital pathogens and adverse pregnancy outcomes in sub-Saharan Africa. The results will provide an evidence base to support screening and management initiatives for non-viral genital pathogens. They will further highlight where there is a need to strengthen public health strategies to continue lowering the burden of non-viral genital pathogens and adverse pregnancy outcomes in sub-Saharan Africa.

## Methods

Searches according to the Preferred Reporting Items for Systematic Review and Meta-Analysis Statement (PRISMA) guidelines were conducted in PubMed/Medline and Embase (Ovid) among studies published up to and including October 2022 (31).

### Search strategy

The following keywords were used: “sexually transmitted infections”, “non-viral”, “adverse pregnancy outcome”, “Africa”, “sub-Saharan Africa”, “pregnant women”, “pregnancy”, and “pregnancy outcome”. To ensure all of the available studies were included, keywords “Africa” or “sub-Saharan Africa” were switched to the individual name of the 48 sub-Saharan African countries (as defined by the World Bank) (32). The Medical Subject Heading (MeSH) and Embase subject heading (Emtree) terms, free-text terms, and combinations of these keywords are compiled in Supplementary Table S1. Lastly, bibliographies of articles with information about non-viral genital pathogens and adverse pregnancy outcomes were examined, even if articles were excluded, to retrieve potential articles via snowballing.

### Inclusion and exclusion criteria

Articles reporting data on the association with non-viral genital pathogens and late pregnancy outcomes (>20 gestational age weeks) were eligible. Early and late pregnancy outcomes were differentiated because of the mechanism of action of non-viral pathogens can vary during the course of pregnancy and can have different influences on the pregnancy and the clinical implications. Prospective and retrospective cohorts, case-control and cross-sectional studies were included, although due to ethical considerations, prospective studies on curable non-viral genital pathogens that did not offer treatment were not expected to be found. The eligibility criteria were: pregnant women from sub-Saharan Africa with known perinatal outcomes; non-viral sexually transmitted infection contracted before or during pregnancy, diagnosed in samples collected before 36 gestational age weeks and not treated; pregnant women who tested negative for the same infection and from the same community as the cases; late pregnancy outcomes and adverse events taking place after 20 weeks. The threshold at 20 GA weeks was used following existing conventional definitions for stillbirth (33). Studies were excluded if infected women received treatment intrapartum, if the total amount of women investigated in the exposure and control group was not stated, if participants were tested for non-viral genital pathogens right before, during or after delivery, and if no control group was included. Study designs that were excluded from this review were (systematic) reviews, case reports, and case series or (conference) abstracts, however if found applicable, the reference list was searched to retrieve possible eligible article. There was no restriction on the microbiological methods used for pathogen detection, but articles were excluded if they did not explicitly provide information on the detection method.

### Study selection

Articles reporting different outcomes for the same cohort were included, if the same cohort and outcomes were reported in multiple manuscripts, the manuscript with the most complete data (often the most recent) was included. Authors CG, NJ, LB and DV independently performed the literature search. Conforming to the PRISMA guidelines, firstly titles or abstract were screened. Relevant publications were identified for full text read and were assessed based on inclusion and exclusion criteria as previously mentioned. Articles were discussed among authors in case there were any doubts regarding eligibility. If no consensus could be reached, articles were sent to author EA for further evaluation regarding their eligibility.

### Data extraction

Country, population characteristics, number of pregnant women included per group, gestational age at testing, pregnancy outcomes, and the unadjusted and adjusted Odds Ratio (OR) and 95% Confidence Interval (CI) were extracted or calculated (based on the dichotomous data given) from each included manuscript. The following late adverse pregnancy outcomes, following the World Health Organisation definitions, were selected: preterm birth (<37 weeks 0 days gestation) (34), low birth weight (<2,500 g), term birth with low birthweight (≥37 weeks 0 days gestation and <2,500 g) (35), intra-uterine growth restriction (infants with birth weight below the 10th percentile for gestational age) (36), spontaneous miscarriage or stillbirth (delivery of stillborn infant ≥20 weeks 0 days) (37) and neonatal death (death within first 28 days of life) (38).

### Risk of bias within studies

The methodological quality of each included study was assessed following the guidelines of the Joanna Briggs Institute reviewer's manual (39). In particular, the guidelines for quality analysis of cohort studies and case control studies were used. The questions used for quality assessment can be found in the Supplementary Table S2*.* Based on a list of critical appraisal questions specific per study design, the percentage of the risk of bias was calculated for every included paper. Every question was answered with “yes”, “no” or “unclear”. For “yes” 1 point was assigned, for “no” 0 points and for “unclear” 0.5 points (39–41). The total amount of points divided by the number of questions multiplied by 100 gave the percentage of risk of bias. A score of 70% or higher indicated a low risk of bias, a score between 50%–69% indicated a moderate risk of bias and a score below 50% a high risk of bias (39–41). The quality assessment was performed by CG, NJ and DV independently and was discussed afterwards, as recommended by the Joanna Briggs Institute (39).

### Data analysis

Dichotomous outcome data extracted from individual studies were used to generate OR, irrespective of study quality. Forest plots, depicting ORs were performed using RevMan. Meta-analysis was conducted when two or more studies with the same study design reported the same outcome, calculating summary statistics from each study, followed by the calculation of summary combined intervention, as weighted average.

## Results

### Study selection

**Figure 1:**
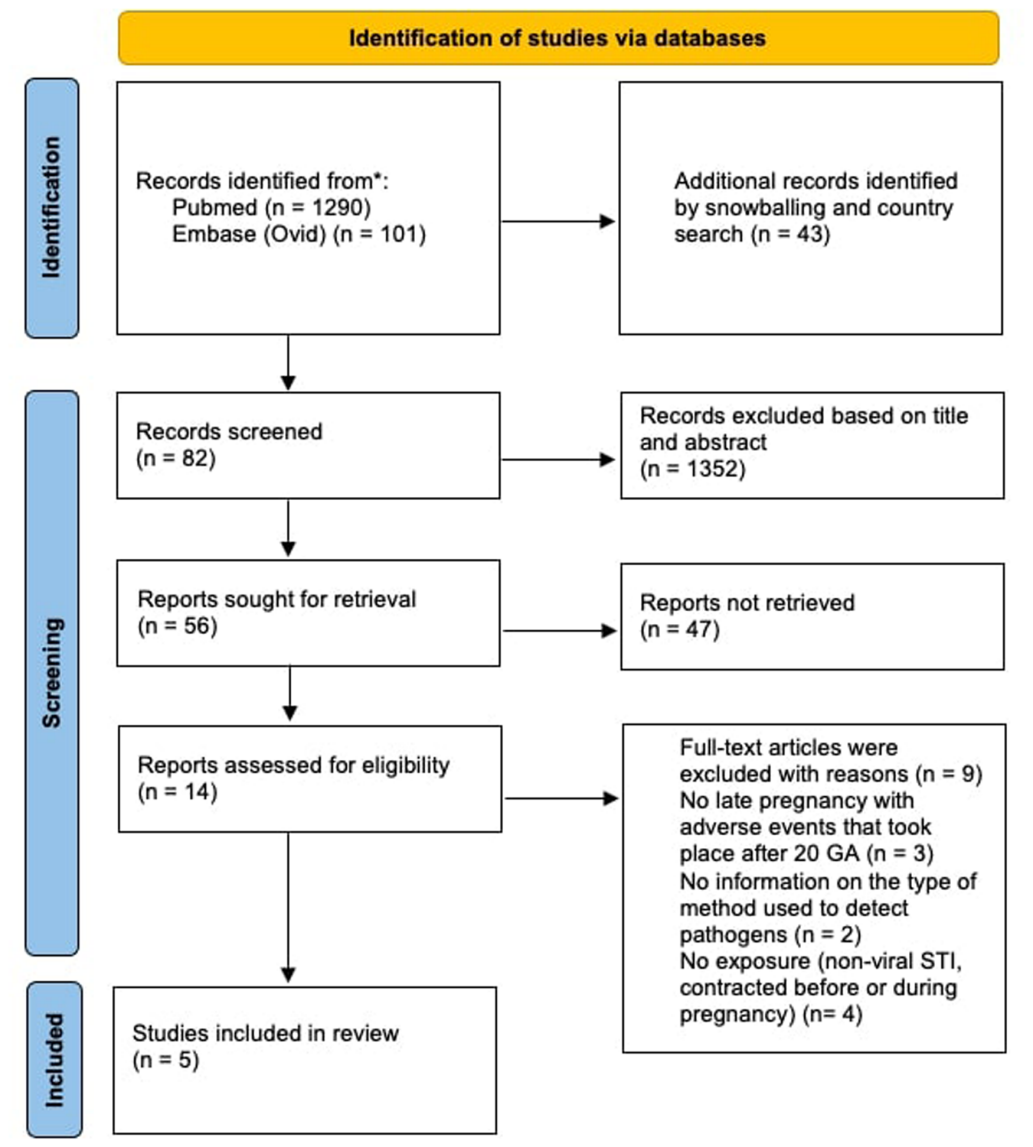
PRISMA flow diagram.

The search strategy used in this review yielded 101 records from Embase, 1,290 from PubMed and 43 through snowballing and individual country search. Among them, five met the inclusion criteria (42–46). The study selection process is shown in the PRISMA flow diagram (Figure 1). The selected studies reported pregnant women living in sub-Saharan Africa, experiencing adverse late pregnancy outcomes in relation to non-viral pathogens.

### Characteristics of the included studies

In total, two retrospective cohort studies, one cohort study and two cross-sectional studies from four sub-Saharan African countries were included. One cohort of pregnant women was based in South Africa, two in Malawi, one in Tanzania and one in Ethiopia. A summary of the cohort characteristics and methodological features of included studies is provided in Table 1. Four studies used venous blood samples for detection of *Treponema pallidum*. They used the rapid plasma reagin (RPR) test, of which Watson-Jones et al. (43) followed up with a specific *T. pallidum* hemagglutination assays and, as a confirmatory test, indirect fluorescent antibody technique. McDermott et al. (42) coupled the RPR test with a *T. pallidum* micro hemagglutination assay as a confirmatory test. One study used endocervical swabs for detection of *C. trachomatis* and *Candida Albicans*. Tareke et al. (44) used Immunochromatography test strips as a confirmatory test beside the RPR. Van Rensburg and colleagues (46) used a commercial enzyme immunoassay for the detection of *C. trachomatis*.

**Table 1:** Summary of the cohort characteristics and methodological features of retrieved studies.

Author, year	Country	Study design	Study population	Follow Up	Methods to detect pathogens	Number of participants	Maternal age (years)	Gravidity/parity	Other infections	Pregnancy outcome	N women adverse pg. outcomes	N women with genital infections + adverse pg. outcome	Odds ratio	*P* value
D Watson-Jones et al., 2002	Tanzania	Retrospective cohort study	Pregnant women, indication of multiple gestation or pre-eclampsia	5 days after birth	RPR, THPA, FTA-ABS, AMPLICOR CT/NG PCR assay	(+) 73 high titer active & 27 low titer active	24,5	35% primigravidae, 14% secundigravidae, 52% multigravida	*Chlamydia trachomatis n* = 18, *Neisseria gonorrhoeae n* = 5	Stillbirth = dead foetus of >22 GA	21/306 = 6,86%	18/73	OR = 25.09	95% CI 7.14-88-21
						(-) 233			HIV *n* = 11, *n* = 7 in high titer active group and *n* = 4 in seronegative group (*p* = 0.3)					*P* < 0.0001
									* *	LBW = birthweight <2,500 g	LBW = 41/255 = 16,08%	18/55	OR = 4.63	95% CI 2.27–9.45
									* *	PTB = delivery at <37 GA	PTB = 18/255 = 7,06%	11/55	OR = 7.96	95% CI 2.93–21.68
									* *	IUGR = birth weight < 2,500 g at ≥37 GA	IUGR = 23/255 = 9,02%	7/55	OR = 1.95	95% CI 0.76–5.00
J McDermott et al., 1993	Malawi	Retrospective cohort study	HIV-negative pregnant women with singleton pregnancy	Every 2 months at least one year after delivery	VDRL or RPR and MHA-TP	(+) 130	Not given	Singleton pregnancy	No	Foetal loss	130/3,015 = 4,31%	30/125	OR = 8.81	5.41–14.29
						(−) 2,968								
									* *	Stillbirth = no definition given	99/2,984 = 3,32%	27/122	OR = 11.01	6.54–18.49
									* *	Macerated stillbirth = no definition given	21/2,906 = 0,72%	8/103	OR = 18.07	6.62–48.41
									* *	Fresh stillbirth = no definition given	30/2,915 = 1,03%	6/101	OR = 7.34	2.60–19.64
									* *	Unknown type of stillbirth = no definition given	48/2,933 = 1,64%	13/108	OR = 10.91	5.25–22.34
									* *	Neonatal death = death within the first 28 days of life	135/2,858 = 4,72%	16/95	OR = 4.50	2.43–8.23
									* *	Early neonatal death = death within the first 7 days of life	91/2,859 = 3,18%	12/95	OR = 4.91	2.42–9.77
									* *	Late neonatal death = death within the days 8 and 28 of life	44/2,767 = 1,59%	4/83	OR = 3.35	0.98–10.18
VJ van Rensburg, HJ Odendaal, 1991	South Africa	Cohort study	Pregnant women who had not received antibiotics in the previous 3 months	Till 28 days postpartum	Chlamydiazynme swab	(+) 20	25	3,7	*U. urealyticum*, 39%	Preterm labour = not defined	36/180 = 20%	8/17	OR = 4.29	1.52–12.07
						(−) 160			*M. hominis,* 20%					
									*C. albicans*, 7%					
									*N. gonorrhoeae*, 6%					
									group B streptococcus, 1%					
Abrams et al., 2004	Malawi	Cross-sectional study	All pregnant women latest phase of labour	After delivery	Hemocue AB, RPR, Serocard rapid HIV test	(+) 55		33.0% (186 of 572) were primigravid	HIV, Maternal malaria	PTB (<36 weeks)	47/522 = 9%	7/47	OR: 9,22	3.2605–26.0532
						(−) 514								
Tareke et al., 2019	Ethiopia	Cross-sectional study	Pregnant women	Not stated	RPR and ICS as a confirmatory test	(+) 10	26,95	43,5% one pregnancy, 30,7% two pregnancy, 25,8% more than 2		Stillbirth = dead foetus of >22 weeks GA	(95) 24,7%	10	OR: 1,25	CI: 0,46–3,41
						(−) 374								

RPR, Rapid plasma regain; THPA, Treponema pallidum hemagglutination assays; FTA-ABS, indirect fluorescent antibody technique; LBW, Low birth wight; PTB, Preterm birth; IUGR, Intrauterine growth restriction; VDRL, Venereal Disease Research Laboratory; MHA-TP, Treponema pallidum micro hemagglutination assay; ICS, Immunochromatography test strips.

#### Preterm birth

**Figure 2:**
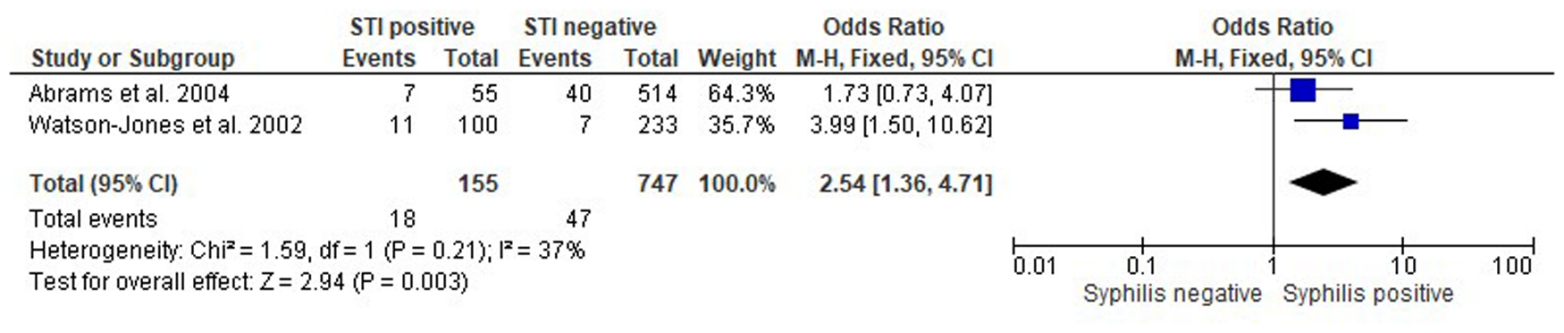
Forest plot showing the odds ratios, confidence intervals and *P*-value for interaction of Treponema pallidum infection and preterm birth in pregnant people.

Two studies have investigated the association between *T. pallidum* infection and PTB. Watson-Jones et al. (43) used the traditional cutoff value of birth at gestational age <37 weeks to define PTB. Abram and colleagues (45) have defined PTB using a cutoff at 36 gestational weeks. Watson-Jones and colleagues have reported a significant association between syphilis and PTB, recording a 4-fold increase in the incidence of PTB in women infected with *T. pallidum* infection compared to non-infected women (CI: 1.50–10.62). Similarly, Abrams et al. have recorded that women with *T. pallidum* infection were 1.73 times more likely (CI: 0.73–4.07) to experience PTB (Figure 2).

In their study based in South Africa, Van Rensburg et al. (46) investigated the association between *C. trachomatis* infection and PTB, defined as the delivery before 37 weeks of gestation. The authors reported a statistically significant increase in incidence of PTB attributable to a positive *C. trachomatis* infection diagnosis (OR: 4.29, CI: 1.52–12.07).

**Figure 3:**
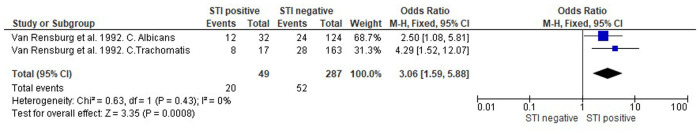
Forest plot showing the odds ratios, confidence intervals and *P*-value for interaction of Chlamydia trachomatis infection and preterm birth & Candida albicans infection and preterm birth in pregnant people.

Additionally, Van Rensburg and colleagues investigated the association between PTB and C. *albicans* infection. The authors found a positive association between the two, reporting a 2.50-fold increase of PTB among *C. albicans* positive women compared to *C. albicans* negative (1.08–5.81) (Figure 3).

#### Stillbirth

**Figure 4:**
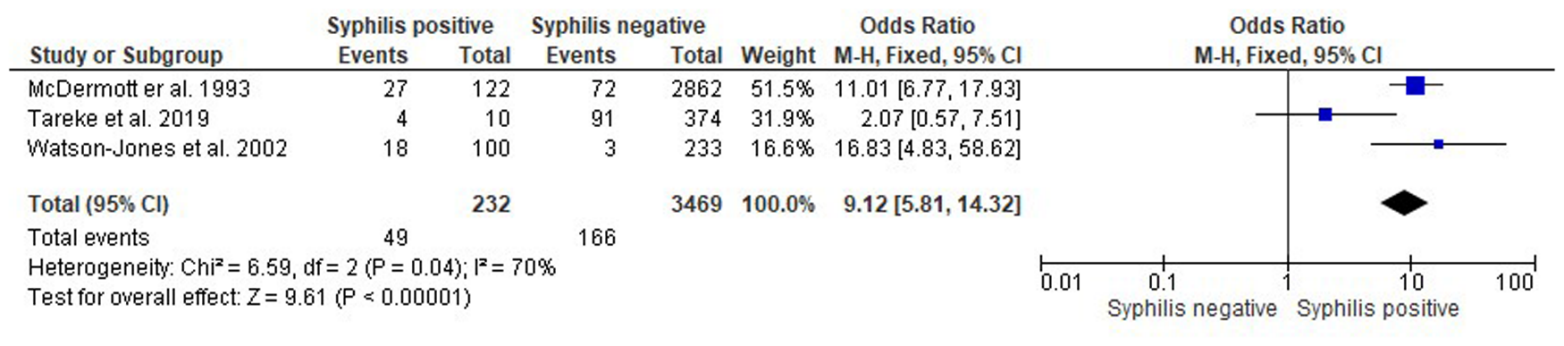
Forest plot showing the odds ratios, confidence intervals and *P*-value for interaction Treponema pallidum infection and stillbirth in pregnant people.

Three studies examined the association between *T. pallidum* infection and stillbirth. Studies by both Watson et al. (43) and Tareke et al. (44) have defined stillbirth as the death or loss of the baby before 22 weeks of gestation. McDermott et al. (42) did not have the same definition, and divided stillbirth into three categories, macerated, fresh and unknown instead. All three studies conducted in Tanzania, Malawi and Ethiopia, have reported a positive association between *T. pallidum* infection and stillbirth with OR ranging from 2.07–16.83 (Figure 4).

#### Neonatal death

**Figure 5:**
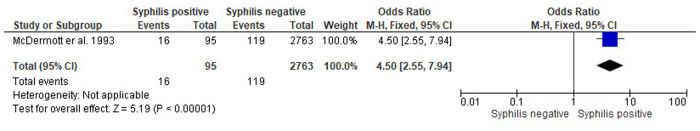
Forest plot showing the odds ratios, confidence intervals and *P*-value for interaction Treponema pallidum infection and neonatal death in pregnant people.

McDermott et al. (42) have investigated the association between *T. pallidum* infection and neonatal death. The authors have defined neonatal death as the death of a newborn in the first 28 days, and they further divided this into early death (first 7 day of life) and late death (day 8–28). A significant association was found between *T. pallidum* infection and neonatal death. Based on this study, pregnant women in Malawi who are infected with *T. pallidum* have a 4.50-fold increase in the incidence of neonatal death, compared to non-infected mothers (CI: 2.55–7.94) (Figure 5).

#### Low birthweight

**Figure 6:**
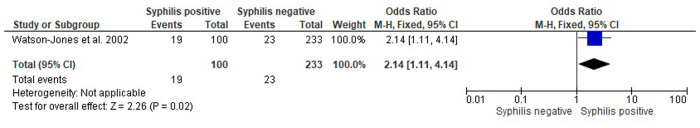
Forest plot showing the odds ratios, confidence intervals and *P*-value for interaction Treponema pallidum infection and low birth weight in pregnant people.

The study by Watson-Jones *et al*. (43) set in Tanzania investigated the association between *T. pallidum* infection and LBW. They defined a LBW infant as one born weighting 2,500 grams or less. The authors have reported a significant association between *T. pallidum* infection and LBW, in particular a 2.14-fold increase in incidence (CI 1.11–4.14) of LBW attributable to a positive infection (Figure 6).

#### IUGR

**Figure 7:**
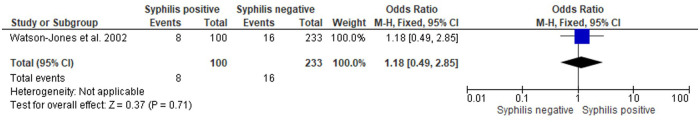
Forest plot showing the odds ratios, confidence intervals and *P*-value for interaction Treponema. pallidum infection and intrauterine growth restriction in pregnant people.

Lastly, Watson-Jones and colleagues (43) have also reported the association between *T. pallidum* infection and IUGR in Tanzania. Defined as a fetus whose estimated weight is below 2,500 grams with a gestational age of ⩾37 weeks, the authors found a 1.18-fold increase in incidence of IUGR (CI: 0.49–2.85) among the *T. pallidum* positive population (Figure 7).

## Discussion

The purpose of the current study was to review the evidence on the association between non-viral genital pathogens and adverse pregnancy outcomes in sub-Saharan Africa. This study comprehensively and systematically identified and reviewed published data on the association between infections by *T. pallidum*, *C. trachomatis* and *C. albicans* and PTB, stillbirth, neonatal death, LBW and IUGR in the region.

Syphilis has long been known to be an important risk factor for adverse pregnancy outcomes, with data from LMICs confirming that maternal syphilis remains an extremely important cause of perinatal morbidity and mortality (24, 47–51). The natural history of syphilis acquired in pregnancy is believed to follow the sequential stages of primary, secondary, and latent syphilis that have been observed in untreated, non-pregnant adult cases. Left untreated, syphilis has a dramatic impact on pregnancy outcome. The consequences of untreated maternal infection include stillbirth, LBW, PTB and IUGR, as shown in this review, also congenital infection in a proportion of surviving infants and neonatal death (52, 53).

Prevalence of syphilis among pregnant women is high in sub-Saharan Africa, particularly in the Southern and East African regions. Rates fell from 2012–2016, indicating sustained progress toward syphilis elimination, although they remain far from the World Health Organisation target of 90% reduction of the incidence of *T. pallidum* infections globally by 2030 (54). This trend can be observed globally, however, with increase in syphilis incidence in the Americas and the Eastern Mediterranean Regions (55). Improvements in access to antenatal care must be hastened, all women accessing antenatal care must be checked for syphilis early in pregnancy and treated correctly, and efforts to test and treat partners and higher-risk groups must be broadened to lower overall community prevalence levels.

Similarly, the burden of chlamydia remains an overlooked global health issue, particularly in LMICs and areas with limited resources. The consequences are exacerbated for pregnant women, where infection may be detrimental to the health of both mothers and their infant. In fact, over the past few decades, multiple studies have demonstrated that chlamydia in pregnancy may lead to adverse pregnancy outcomes including miscarriage, PTB and stillbirth through placental damage or direct fetal infection (28, 56, 57). The worldwide burden of non-viral genital pathogens like *C. trachomatis* is felt most intensely by women in LMICs, with the repercussions for pregnant women amplified even more, posing concerns to maternal and child health. International prevalence studies of *C. trachomatis* infection in pregnant women reveal similar, if not greater, prevalence rates than in nonpregnant women (52). Individual studies of pregnant women in Sub-Saharan Africa suggest prevalence rates of 0–31.1%, while pooled prevalence rates are 6.9% in East and Southern Africa and 6.1% in West and Central Africa. Among Sub-Saharan African reproductive age women, pooled prevalence of Chlamydia was 7.8% (28). The study included in this review and conducted in Tanzania is consistent with other research and confirms that chlamydia is associated with PTB. *C. trachomatis* infection can induce the inflammation of the upper genital tract, and during pregnancy its ascension can extend into placental tissues (58–60). Such an infection of placental tissues tends to trigger inflammatory reactions, subsequently contributing to PTB. The findings support implementation of screening and treatment for chlamydia during pregnancy in women who are at a high risk of contracting it (61). Significant hurdles have historically hampered efforts to enhance worldwide chlamydial screening and treatment practices for pregnant women. Despite improved detection of *C. trachomatis* infection through molecular-based nucleic acid testing, more patient-friendly specimen collection methods and simple, highly effective, one-dose oral treatment regimens are needed, with only a few countries have made chlamydia screening and treatment a priority for pregnant women (62, 63). While certain countries, such as the United States, have advocated for universal chlamydia screening and treatment for all pregnant women since the 1990s, firstly recommending it for women younger than 25 years old, this approach is not standard practice worldwide (28).

The lower rates of *C. trachomatis* infections found in the articles included in this review, compared to those from studies performed in some high-income countries, including the United States, can be explained by different elements (64, 65). Case-notification reports and epidemiological research are main sources of information on the prevalence and incidence of a specific disease. For disease with specific symptoms in countries with effective reporting systems, the number of reported cases is a fair representation for the overall burden of infections. Sexually transmitted infections, on the other hand, are frequently symptomless, and when symptoms occur, they are frequently not specific. The estimates pooled for this review, while based on a comprehensive examination of available information, are limited by the amount and quality of data available from different locations, as well as our understanding of pathophysiology infection. The nature of the populations tested, small sample numbers, and the diverse diagnostic techniques further influence results (66).

Furthermore, testing techniques vary substantially and approaches, such as nucleic acid amplification tests, which has been proven to be more sensitive and specific, compared to enzyme immunoassay tests, may require transport to specialize laboratory, as well as specialized equipment and staff, delaying the availability of results for rapid management choices in certain regions (67). As a result, in some cases, the most accurate test may not be the approach eventually used.

Additionally, the frequency and incidence of sexually transmitted infections vary greatly across countries, even with among similar population and might reflect several social, cultural, and economic variables, as well as access to adequate treatment (68).

Since syphilis and chlamydia are easily curable infections, antenatal screening programs that identify and treat infected mothers could potentially prevent many of the associated pregnancy and neonatal complications. It is fundamental to take into consideration that given the existing healthcare system constrains of many countries in Sub-Saharan Africa, diverse populations and different healthcare necessities, novel technologies should be designed strategically for local implementation (69). Ensuring appropriate treatment and diagnosis of non-viral genital pathogens in mothers should be a priority for local governments and health ministries, reminding policy-makers that the elimination of such infections is a public health priority that can enable progress towards the Sustainable Development Goal 3 (to ensure healthy lives and promote well-being for all at all ages); especially because of the health and financial burden and consequences it has for the mother and child and ultimately the population. Moreover, the challenges in infrastructural set-up and economic structures are aggravated by the fact that non-viral genital pathogens do not occur in isolation, but as co-infections with similar and unspecific symptoms (52). In resource limited settings, control efforts range from symptoms-based epidemiologic treatment with no diagnostic test support, to laboratory-based screening of symptomatic and asymptomatic people using molecular technologies (70). One of the issues that emerges from the results of this review is that there is an urgent need for affordable, rapid, point-of-care tests (POCTs) for non-viral genital pathogens screening in resource constrained antenatal care settings. Rapid testing and prompt clinical decision can contribute to better maternal and neonatal health outcomes, especially in resource constrained settings. Consistent with previous literature that called for implementation of treponemal or non-treponemal POCTs in antenatal care settings in which pregnant women receive no screening (71), the data collected in this review could be considered a call to action for health systems integration of fast, accessible and affordable diagnostics tools, such as POCTs, in order to mitigate the transmission and burden of genital pathogens, which affects women disproportionally.

*C. albicans*, an opportunistic pathogenic yeast, is the most frequent microorganisms responsible for different problems, such as vulvovaginal candidiasis, a common vaginal dysbiotic condition (72). This pathogenic yeast is commensal to the human body. Increased levels of progesterone and estrogen in women during pregnancy impact the vaginal microenvironment (vaginal microbiota) and can contribute to the acquisition of genital infections (73–75). The risk of candidiasis has been reported to rise during pregnancy, especially in the third trimester, increasing the risk of pregnancy-related problems such as PTB and LBW (76). In fact, recent research suggests that abnormal vaginal microbiota are associated with adverse pregnancy outcomes (77, 78).

Multiple studies have evaluated the prevalence of vulvovaginal candidiasis among pregnant and nonpregnant women and shown that pregnant women had a greater prevalence rate than nonpregnant women (77). The frequency of vulvovaginal candidiasis among pregnant women worldwide ranged from 17%–90% and is more common among pregnant women in Asian and African nations. Although *C. albicans* has been found to be responsible for the majority of vulvovaginal candidiasis-related symptoms in numerous studies worldwide, multiple Asian and African countries have shown an increase in the detection rate of non-albicans *Candida spp*., as well as of infections caused by them over the last three decades. Numerous investigations have indicated that infections by non-albicans *Candida spp*. outnumber those by *C. albicans* (79–81). *Candida spp*. should be regarded as potentially dangerous pathogens in the early stages of pregnancy. Granting the fact that reducing adverse pregnancy outcomes is a multi-factorial success with multiple variables, our findings suggest that regular candidiasis screening and subsequent treatment might help improve pregnancy outcomes.

Currently, as nations devote resources to the control of health emergencies, genital infections services are available only to symptomatic patients in many settings, and these patients are frequently managed syndromically, based on regional epidemiology, genital infections testing supply and staff shortages (82). Since POCT can be a game changer in increasing healthcare quality for the entire population, it is imperative for stakeholders and policy makers to identify innovative POCT that can show maximal potential to revive the response to genital infections across the country. The importance of such technologies is also highlighted recently by the COVID-19 pandemic, where such tools play a critical role in the containment of the pandemic (83, 84).

This study is subject to several limitations. Because of availability of treatment and ethical reasons, most of the included studies are scant and rather dated. Nonetheless, it remains important to review the potential burden of infections by non-viral genital pathogens during pregnancy because non-symptomatic untreated infections continue having consequences for the pregnancy outcome. It should also be mentioned that some of the studies included did not control for confounding factors, such as Human Immunodeficiency Virus status. As the confounding factor analysis was not performed in this study thus there could be an overestimation of the odds ratios obtained in the pooled data. Finally, one source of weakness in this study, is that the methodology and the analysis that were used in the retrieved publications might have low sensitivity, resulting in low prevalence of non-viral pathogens. Laboratory-based nucleic acid amplification tests are accurate diagnosis but often not affordable or accessible in resource limited settings due to the high cost of laboratory infrastructure and trained staff.

## Conclusion

This study provides an overview of the association of non-viral genital infections and adverse pregnancies outcomes in sub-Saharan Africa. An increased risk of PTB has been associated with *Treponema pallidum, C. trachomatis* and *C. albicans* infections in Tanzania and South Africa, respectively*.* Additionally, rates of stillbirth, neonatal death, LBW and IUGR have been associated with *T. pallidum* infection Tanzania, Malawi and Ethiopia in independent studies*.* The evidence provided here is in agreement with results of other world regions. The data summarized in the review, combined with the latest data from prevalence studies conducted in sub-Saharan Africa, is a reminder that interventions are still needed to increase the prevention and treatment of non-viral genital pathogens and ultimately improve maternal and neonatal health. It is also a call for action to develop cost-effective and inclusive public health programs for treatable non-viral genital infections in sub-Saharan Africa, including in remote areas. Countries in this region should continue to reevaluate their screening programs, diagnostics methods and treatment criteria to lower the burden associated these treatable non-viral pathogens.

## Data Availability

The original contributions presented in the study are included in the article/**Supplementary Materials**, further inquiries can be directed to the corresponding author.
