# Supplementary material for: The association between adverse pregnancy outcomes and non-viral genital pathogens among women living in sub-Saharan Africa: a systematic review
Source: Front Reprod Health. 2023 Jun 7;5:1107931. doi: 10.3389/frph.2023.1107931 (PMC10282605; doi:10.3389/frph.2023.1107931)
Supplement: Supplementary file 1 [file Table1.docx]

Supplementary Table 1. Search strategies and hits based on searches last conducted on October 2022.

| **Database** | **Search strategy** |
| --- | --- |
| Pubmed | (("Reproductive Tract Infections"[Mesh]) OR ("Sexually Transmitted Diseases"[Mesh]) OR (genital tract infection) OR (reproductive tract infection) OR (sexually transmitted infection) OR (sexually transmitted disease) OR (vaginal infection) OR (cervical infection)) AND (("Pregnant Women"[Mesh]) OR ("Pregnancy"[Mesh]) OR (pregnancy) OR (pregnant women)) AND (("Pregnancy Outcome"[Mesh]) OR (adverse pregnancy outcome) OR (pregnancy outcome)) AND (("Africa"[Mesh]) OR ("Africa South of the Sahara"[Mesh]) OR (Sub-Saharan Africa) OR (Africa) OR (African women)) |
| Embase (Ovid) | (exp Reproductive Tract Infections/ or exp Sexually Transmitted Diseases/ or exp genital tract infection or (reproductive tract infection or sexually transmitted infection or sexually transmitted disease or vaginal infection or cervical infection).ti,ab,kw. AND exp africa/ or exp Africa south of Sahara/ or sub-Saharan Africa.ti,ab,kw. AND exp pregnant women/ or exp pregnancy/ or (pregnant or pregnancy).ti,ab,kw. |
