# Supplementary material for: The association between adverse pregnancy outcomes and non-viral genital pathogens among women living in sub-Saharan Africa: a systematic review
Source: Front Reprod Health. 2023 Jun 7;5:1107931. doi: 10.3389/frph.2023.1107931 (PMC10282605; doi:10.3389/frph.2023.1107931)
Supplement: Supplementary file 2 [file Table2.docx]

Supplementary Table 2. Questions for the critical appraisal checklist for cohort studies and cross sectional studies, trial as provided by Joanna Briggs Institute Reviewer’s Manual.

| **Question numbers** | **Cohort studies** | **Cross-sectional studies** |
| --- | --- | --- |
| 1 | Were the two groups similar and recruited from the same population? | Were the criteria for inclusion in the sample clearly defined? |
| 2 | 1. Were the exposures measured similarly to assign people to both exposed and unexposed groups? | Were the study subjects and the setting described in detail? |
| 3 | Was the exposure measured in a valid and reliable way? | Was the exposure measured in a valid and reliable way? |
| 4 | Were confounding factors identified? | Were objective, standard criteria used for measurement of the condition? |
| 5 | Were strategies to deal with confounding factors stated? | Were confounding factors identified? |
| 6 | Were the groups/participants free of the outcome at the start of the study (or at the moment of exposure)? | Were strategies to deal with confounding factors stated? |
| 7 | Were the outcomes measured in a valid and reliable way? | Were the outcomes measured in a valid and reliable way? |
| 8 | Was the follow up time reported and sufficient to be long enough for outcomes to occur? | Was appropriate statistical analysis used? |
| 9 | Was follow up complete, and if not, were the reasons to loss to follow up described and explored? | Not applicable |
| 10 | Were strategies to address incomplete follow up utilized? | Not applicable |
| 11 | Was appropriate statistical analysis used? | Not applicable |
| 12 | Not applicable | Not applicable |
| 13 | Not applicable | Not applicable |
